# Cycle exercise training and muscle mass: A preliminary investigation of 17 lower limb muscles in older men

**DOI:** 10.14814/phy2.15781

**Published:** 2023-08-22

**Authors:** Masatoshi Naruse, Caroline S. Vincenty, Adam R. Konopka, Scott W. Trappe, Matthew P. Harber, Todd A. Trappe

**Affiliations:** ^1^ Human Performance Laboratory Ball State University Muncie Indiana USA

**Keywords:** aging, cycling, exercise, sarcopenia, skeletal muscle mass

## Abstract

Cycling exercise in older individuals is beneficial for the cardiovascular system and quadriceps muscles, including partially reversing the age‐related loss of quadriceps muscle mass. However, the effect of cycling exercise on the numerous other lower limb muscles is unknown. Six older men (74 ± 8 years) underwent MRI before and after 12‐weeks of progressive aerobic cycle exercise training (3–4 days/week, 60–180 min/week, 60%–80% heart rate reserve, *V*O_2_max: +13%) for upper (rectus femoris, vastii, adductor longus, adductor magnus, gracilis, sartorius, biceps femoris long head, biceps femoris short head, semimembranosus, semitendinosus) and lower (anterior tibial, posterior tibialis, peroneals, flexor digitorum longus, lateral gastrocnemius, medial gastrocnemius, soleus) leg muscle volumes. In the upper leg, cycle exercise training induced hypertrophy (*p* ≤ 0.05) in the vastii (+7%) and sartorius (+6%), with a trend to increase biceps femoris short head (+5%, *p* = 0.1). Additionally, there was a trend to decrease muscle volume in the adductor longus (−6%, *p* = 0.1) and biceps femoris long head (−5%, *p* = 0.09). In the lower leg, all 7 muscle volumes assessed were unaltered pre‐ to post‐training (−2% to −3%, *p* > 0.05). This new evidence related to cycle exercise training in older individuals clarifies the specific upper leg muscles that are highly impacted, while revealing all the lower leg muscles do not appear responsive, in the context of muscle mass and sarcopenia. This study provides information for exercise program development in older individuals, suggesting other specific exercises are needed for the rectus femoris and adductors, certain hamstrings, and the anterior and posterior lower leg muscles to augment the beneficial effects of cycling exercise for older adults.

## 1 INTRODUCTION

Older individuals have been shown to be highly responsive to aerobic exercise training to increase exercise capacity (Fujimoto et al., 2010; Harber et al., 2012; Harber, Konopka, et al., 2009; Ogawa et al., 1992). This is beneficial for several reasons, namely that exercise capacity is a strong predictor of mortality (Harber et al., 2017; Imboden et al., 2018; Laukkanen et al., 2022; Myers et al., 2002). Cycling is one mode of aerobic exercise that is promoted for older individuals to increase exercise capacity and overall functionality, through adaptations in the cardiovascular system and within the skeletal muscle (Fritzen et al., 2020; Fujimoto et al., 2010; Harber et al., 2012; Harber, Konopka, et al., 2009; Meredith et al., 1989; Ogawa et al., 1992). Reduction in skeletal muscle mass is also associated with reduced functionality and independence in older individuals (Larsson et al., 2019). While resistance exercise is usually considered the best stimulus for increasing skeletal muscle mass in older individuals (Adams & Bamman, 2012; Lavin et al., 2019), aerobic cycling exercise has also been shown to promote significant increases in quadriceps muscle mass (Konopka & Harber, 2014), the main skeletal muscles involved in this activity.

The quadriceps, comprised of three vastii muscles and the rectus femoris, is an important muscle group of the lower limbs for typical activities of daily living and many recreational activities engaged in by older adults. However, there are numerous other important muscles in the upper and lower leg that also play important roles in gait, balance, and overall functional independence. Investigating only the quadriceps ignores more than 60% of the muscle mass of the lower limbs (Belavy et al., 2009) and ~80% of the specific muscles, all with unique functions. Interestingly, there is a dearth of information on specific muscles of the lower limbs, outside of the quadriceps, in aging (Naruse, Trappe, & Trappe, 2023) or in older individuals in response to exercise training (Chambers et al., 2020; Skoglund et al., 2022).

Metabolic studies provide some insight into understanding cycle exercise for lower limb skeletal muscle health. A large number of studies have shown the vastii muscles are highly metabolically active and adaptive to acute and chronic cycle exercise (Fritzen et al., 2020; Gibala & MacInnis, 2022; Gondoh et al., 2009; Harber et al., 2012; Harber, Konopka, et al., 2009; Konopka et al., 2014; Lester et al., 2013; Meredith et al., 1989; Short et al., 2004; Starling et al., 1997). Advanced imaging techniques coupled with radioactive metabolic tracers have been used to examine muscle‐specific glucose uptake in the upper and lower leg muscles during cycle exercise up to 55% of *V*O_2_max in young men (Gondoh et al., 2009). They show increased glucose uptake in the vastii, rectus femoris, sartorius, biceps femoris, semitendinosus, semimembranosus, adductor magnus, adductor longus, and gracilis; suggesting these muscles would have beneficial metabolic adaptions to chronic cycle training. Interestingly, the lower leg muscles either did not have any (medial and lateral gastrocnemius, soleus) or had a relatively small (tibial muscles) increase in glucose uptake while cycling at 55% of *V*O_2_max. In support of this, we have shown that cycling for 45 min at 55% of *V*O_2_max did not elicit any intramuscular change (i.e., use) in glycogen or triglycerides in the soleus (Lester et al., 2013). It appears that higher‐intensity interval training may be necessary to engage the lower leg muscles and impact lower leg muscle health with cycle training in older (or younger) individuals (Harridge et al., 1998; Lester et al., 2013).

Electromyographic (EMG) studies of cycling using surface EMG of the superficial muscles and fine wire recordings of the deep muscles generally support the aforementioned metabolic studies (Ericson et al., 1985; Gregor et al., 1991; Hug & Dorel, 2009). That is, most of the upper leg muscles show muscle activation at some phase of the pedal revolution, with an increased involvement with increasing workload. However, many of the lower leg muscles are also active, albeit to a relatively small degree in some muscles. The basis of the discrepancy between the lower leg muscle EMG and aforementioned metabolic data is not clear. However, it is known that the various muscles of the upper and lower leg have different underlying phenotypic profiles based on different normal daily activity patterns, which, in turn, may influence the muscle‐specific exercise training response (Chambers et al., 2020; Luden et al., 2012; Trappe, Raue, & Tesch, 2004).

The goal of the current investigation was to examine the volume of all of the skeletal muscles of the lower limb in response to an aerobic cycle exercise training program in older men using magnetic resonance imaging (MRI) of the upper and lower leg. This included an examination of 17 lower limb muscles combined with a 12‐week, laboratory‐based training program that involved one‐on‐one supervised heart‐rate‐based aerobic training sessions.

## 2 MATERIALS AND METHODS

### 2.1 Subjects and overall study design

Six older men volunteered to participate in this investigation (Table 1). Each subject provided a detailed medical history and underwent a thorough physical examination, which included a blood chemistry profile, pulmonary function test, and resting and exercise electrocardiograms. Subjects were excluded based on the following criteria: (1) body mass index ≥28 kg/m^2^; (2) type 1 or type 2 diabetes; (3) uncontrolled hypertension; (4) active cancer, cancer in remission, or having received treatment for any form of cancer in the previous 5 years; (5) coronary artery disease; (6), cardiovascular disease (e.g., peripheral arterial disease, peripheral vascular disease); (7) abnormal thyroid function; (8) engaged in regular aerobic or resistance exercise more than one time per week for 20 min or longer during the previous year; (9) chronic and/or regular nonsteroidal anti‐inflammatory drug consumption; (10) any condition that presents a limitation to exercise training (e.g., severe arthritis, chronic obstructive pulmonary disease, neuromuscular disorder, moderate or severe cognitive impairment, Alzheimer's disease, vertigo, dizziness). Four subjects were on cholesterol‐lowering medications (i.e., statins), three were on blood pressure medications (non‐β‐blocker), and five were on medications for prostate health. Written, informed consent was obtained from each subject for all procedures following approval by the Institutional Review Board of Ball State University.

**TABLE 1:** Subject characteristics and aerobic capacity before and after the 12 weeks of aerobic cycle exercise training.

Characteristic	Pre	Post
Age, years	74 ± 8	‐
Height, cm	177 ± 4	‐
Weight, kg	82.3 ± 10.4	81.5 ± 6.9
BMI, kg/m^2^	26.1 ± 3.3	25.8 ± 2.8
*V*O_2_max, L/min	1.77 ± 0.25	1.99 ± 0.29*
*V*O_2_max, mL/kg/min	21.0 ± 2.4	23.9 ± 2.4*
Cycling max workload, watts	143 ± 14	172 ± 23*

*Note*: Data are from all subjects (*n* = 6).

* *p* ≤ 0.05 from pre‐ to post‐training.

**FIGURE 1:**
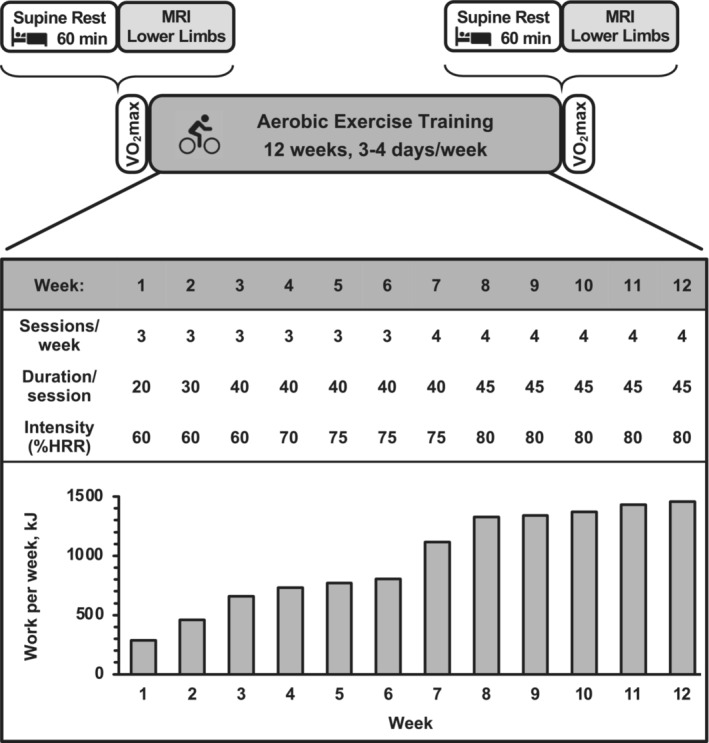
Overall study protocol schematic, including MRI preimaging control and the aerobic cycle exercise training program specifics. There were 42 total exercise training sessions over the 12 weeks. Total exercise time per week ranged from 60 to 180 min, which was 0.6%–1.8% of the available weekly minutes. Work performed on the cycle ergometer per week (kJ) is the average of all subjects and highlights the progressive nature of the training stimulus. HRR, heart rate reserve.

Eligible volunteers underwent measurements of aerobic capacity and whole muscle volume of all the major muscles of the lower limbs assessed with MRI before and after 12 weeks of progressive aerobic cycle exercise training (Figure 1). Data from this investigation have been reported in other publications (Harber et al., 2012; Konopka et al., 2014, 2018).

### 2.2 Aerobic capacity

Subjects performed a graded exercise test for the assessment of maximal aerobic power (*V*O_2_max) before and after the 12‐week aerobic training intervention (Harber et al., 2012). Subjects performed the physician‐supervised test on an electronically braked cycle ergometer (SensorMedics Ergometrics 800) beginning at a very low workload (~10 W), and the workload was progressively increased 10 W in 1‐min stages until exhaustion with a total test time of 10–12 min. During the test, subject's heart rate, blood pressure, rating of perceived exertion, and electrocardiogram were monitored, and ventilation and expired air samples were measured by a metabolic cart (TrueOne 2400 Metabolic System, ParvoMedics) for the determination of O_2_ uptake.

### 2.3 Muscle volume via MRI

**FIGURE 2:**
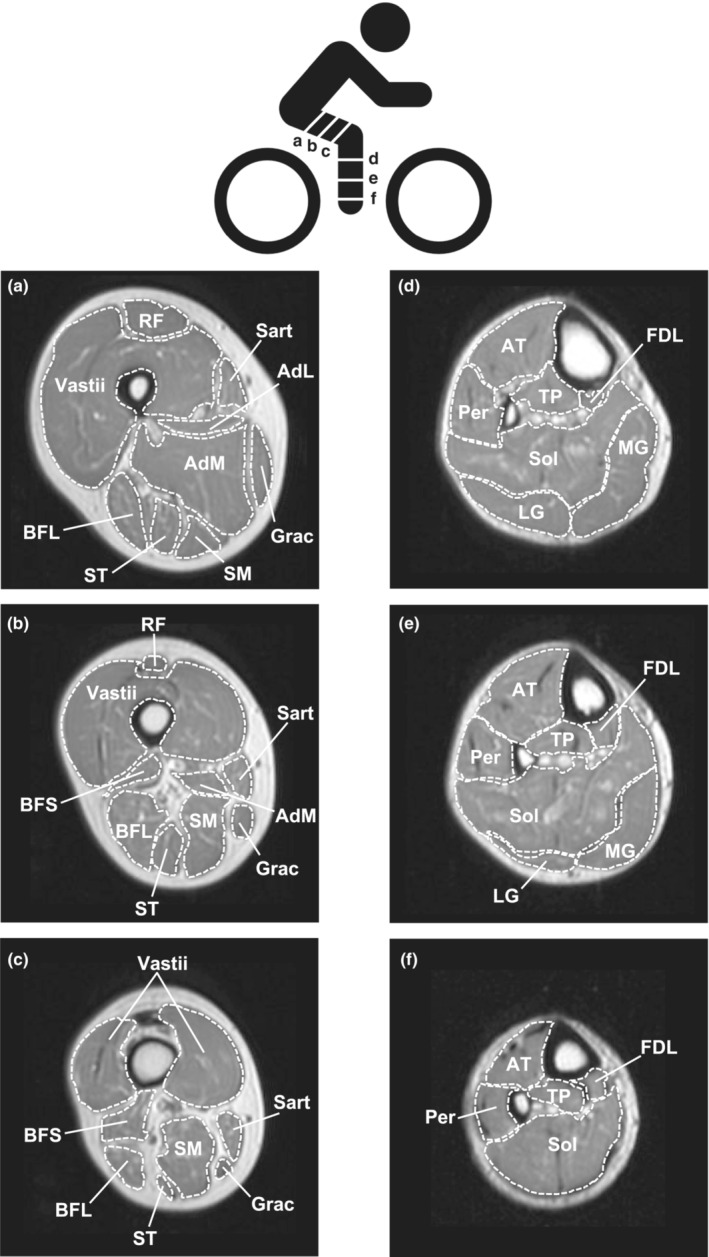
Representative images of three different regions and identification of the 10 upper leg muscles and 7 lower leg muscles studied in the current investigation. AdL, adductor longus; AdM, adductor magnus; AT, anterior tibial muscles; BFL, biceps femoris long head; BFS, biceps femoris short head; FDL, flexor digitorium longus; Grac, gracilis; LG, lateral gastrocnemius; MG, medial gastrocnemius; Per, peroneals; RF, rectus femoris; Sart, sartorius; SM, semimembranosus; Sol, soleus; ST, semitendinosus; TP, tibialis posterior; Vastii, vastus lateralis, vastus intermedius, vastus medialis.

Following 1 h of horizontal, supine rest to control for the influence of postural related fluid shifts on muscle size (Berg et al., 1993), MR imaging was completed on each subject prior to and at the end of the 12‐week exercise training program to determine the volume of the upper (rectus femoris, vastii, adductor longus, adductor magnus, gracilis, sartorius, biceps femoris long head, biceps femoris short head, semimembranosus, semitendinosus) and lower (anterior tibial muscles, posterior tibialis, peroneals, flexor digitorum longus, lateral gastrocnemius, medial gastrocnemius, soleus) leg muscles (Figure 2; Table 2). Specific procedures for the MRI acquisition have been described by us in detail previously (Harber et al., 2012; Trappe, Burd, et al., 2007; Trappe, Lindquist, & Carrithers, 2001). Subjects were supine and their heels were fixed on a nonmetallic foot restraint/positioner to control joint angle (and thus muscle length) and scan angle. Reproducibility across sessions was controlled by the use of the foot restraint and the use of a specific anatomical bony landmark viewed on an initial scout scan for each individual. Subjects also refrained from excessive muscular exercise prior to scanning (Ploutz‐Snyder et al., 1995; Trappe, Burd, et al., 2007). Imaging was completed in a 1.5 T scanner (Symphony, Siemens) with standard settings (repetition time/echo time: 2000/9). Following the scout scan, contiguous interleaved transaxial images of 8 mm thick were taken from the knee to the top of the thigh and the knee to the ankle. MR images were transferred electronically from the scanner to a personal computer and analyzed by the same investigator for all images using manual planimetry.

**TABLE 2:** Contribution of individual muscles to the upper or lower leg and total limb muscle volumes from the older men for general reference.

Muscle	%Upper or lower leg	%Total limb
Upper leg		
Rectus femoris	5	3
Vastii	50	34
Quadriceps	54	37
Adductor longus	4	3
Adductor magnus	17	12
Gracilis	2	1
Total adductors	23	16
Sartorius	2	2
Biceps femoris—long head	6	4
Biceps femoris—short head	3	2
Semimembranosus	7	5
Semitendinosus	4	3
Total hamstrings	21	14
Lower leg		
Anterior tibial muscles	18	6
Peroneals	9	3
Tibialis posterior	7	2
Flexor digitorum longus	2	1
Total anterior muscles	36	11
Lateral gastrocnemius	9	3
Medial gastrocnemius	14	4
Soleus	41	13
Total triceps surae	64	20

*Note*: Data are from pretraining.

The three vastii muscles (vastus lateralis, vastus medialis, and vastus intermedius), three anterior tibial muscles (tibialis anterior, extensor digitorum longus, and extensor hallucis longus), two peroneal muscles (peroneus brevis and longus), and soleus and flexor hallucis longus were circumscribed together, respectively. The adductor brevis and pectineus were not included due to difficulty in differentiation (Belavy et al., 2009). For each muscle or muscle group, the middle 70% of its visible length within the available images was considered for muscle volume determination for the upper and lower leg muscles. Due to the presence of certain muscles that originate or insert outside of the MRI scan, an additional approach was employed for the upper leg muscles. Since the biceps femoris long head consistently appeared in the middle of the thigh for all subjects, its length (biceps femoris long head length; BFLL) was utilized to determine the relative region selection. The process of determining muscle area started at 20%BFLL proximal to the slice where the last visible gluteus muscles were observed and ended at 10%BFLL distal to the slice where the last visible biceps femoris long head was observed. Within this region, the middle 70% of the visible length was considered for the cross‐sectional area measurements for each of the upper leg muscles.

Muscle or muscle group cross‐sectional area was measured in every third slice of the selected region. Muscle area was averaged over five measurements with a coefficient of variation of less than 2% within each image. Muscle volume (cm^3^) was calculated by multiplying the cross‐sectional area of each muscle or muscle group by the appropriate slice thickness. The right limb of each subject was used for all measurements.

### 2.4 Aerobic exercise training program

Subjects performed 12 weeks of aerobic exercise training on a cycle ergometer (Monark Ergomedic 828E) (Figure 1). Exercise intensity was standardized among subjects, relative to their maximal aerobic capacity, by utilizing the heart rate reserve method, as we have previously described (Harber et al., 2012; Harber, Konopka, et al., 2009). The ergometer workload was adjusted for subjects to maintain target heart rate (Polar USA), and subjects exercised at an average pedal frequency between 65 and 85 revolutions/min (RPMs). A total of 42 exercise sessions were planned for each subject. Duration (20–45 min), intensity (60%–80% heart rate reserve), and frequency (3–4 sessions/week) of exercise were progressively increased throughout the 12 weeks to optimize the training response. The training program was identical to the protocol we have previously used in older subjects (Harber, Konopka, et al., 2009). Each exercise session was monitored in its entirety by a member of the investigative team to ensure that the prescribed exercise intensity and duration were obtained. Additionally, subject's body weight was measured and recorded before each exercise session (3–4 times/week). Subjects were counseled to make modifications in dietary intake to maintain body weight, which was effective for all but one subject (see Section 3).

### 2.5 Statistical analysis

Comparisons from pre‐ to post‐training for subject characteristics, *V*O_2_max, and individual skeletal muscle volumes were completed with a paired, two‐tailed *t*‐test. Significance was accepted at *p* ≤ 0.05. Means are presented ± SD. In addition, the number of subjects that would be required to show a significant response to training for each muscle was calculated, considering the difference (*δ*) and standard deviation (*σ*) observed in the current investigation, with an *α* = 0.05 and a *β* = 0.2 (i.e., power of 0.8, 80%) (Table S1; https://doi.org/10.6084/m9.figshare.23509494).

## 3 RESULTS

### 3.1 Exercise compliance and subject characteristics.

Each subject completed all 42 exercise sessions for an exercise compliance of 100%. Average total mechanical work per week for the group increased ~400% from week 1 to week 12 (Figure 1). Cycling cadence during the training period averaged 75 ± 2 RPM. Average body weight was unchanged during the training program (Table 1); however, while five of the subjects were weight stable (−1% to +3% of pretraining body weight), one subject did not maintain his pretraining body weight (−9%, −9.3 kg). As a result, his muscle volume responses were unique and are presented in combination with and separately from the group. Absolute and relative (to body weight) *V*O_2_max was increased (*p* ≤ 0.05) 13% and 14%, respectively, with the training program (Table 1). Maximal workload obtained during the *V*O_2_max test increased (*p* ≤ 0.05) 21% (Table 1).

### 3.2 Muscle volume responses

Muscle volumes before and after the 12 weeks of training are presented in Table 3 (10 upper leg muscles) and Table 4 (7 lower leg muscles). Cycle training induced increases (*p* ≤ 0.05) in volume of the vastii (+7%) and sartorius (+6%) muscles, while there was a trend for an increase in the biceps femoris short head (+5%, *p* = 0.1). There was also a trend for a decrease in volume of the biceps femoris long head (−5%, *p* = 0.09) and adductor longus (−6%, *p* = 0.1). The other 5 muscles of the upper leg did not change (*p* > 0.05) muscle volume pre‐ to post‐training. The 7 muscles of the lower leg did not change (−2 to −3%, *p* > 0.05) muscle volume pre‐ to post‐training.

**TABLE 3:** Muscle volumes of the upper leg before and after the 12 weeks of aerobic cycle exercise training.

Muscle	Pre	Post	%Δ
Rectus femoris	105 ± 11	101 ± 10	−3
Vastii	1154 ± 109	1237 ± 94*	+7
Quadriceps	1259 ± 110	1338 ± 91*	+6
Adductor longus	94 ± 10	88 ± 8**	−6
Adductor magnus	395 ± 62	388 ± 58	−1
Gracilis	42 ± 12	43 ± 10	+3
Total adductors	531 ± 77	519 ± 66	−2
Sartorius	52 ± 14	55 ± 15*	+6
Biceps femoris—long head	145 ± 37	139 ± 35**	−5
Biceps femoris—short head	61 ± 14	65 ± 14**	+5
Semimembranosus	171 ± 35	166 ± 36	−3
Semitendinosus	101 ± 25	100 ± 19	+1
Total hamstrings	479 ± 79	470 ± 70	−2

*Note*: Data are from all subjects (*n* = 6). Pre and post volumes are cm^3^.

* *p* ≤ 0.05 from pre‐ to post‐training

** *p* ≤ 0.1 pre‐ to post‐training

**TABLE 4:** Muscle volumes of the lower leg before and after the 12 weeks of aerobic cycle exercise training.

Muscle	Pre	Post	%Δ
Anterior tibial muscles	188 ± 22	184 ± 19	−2
Peroneals	95 ± 12	94 ± 11	−2
Tibialis posterior	76 ± 16	75 ± 17	−2
Flexor digitorum longus	26 ± 5	25 ± 5**	−3
Total anterior muscles	385 ± 47	378 ± 43	−2
Lateral gastrocnemius	96 ± 37	91 ± 31	−3
Medial gastrocnemius	155 ± 59	150 ± 51	−2
Soleus	450 ± 111	441 ± 103	−2
Total triceps surae	700 ± 197	682 ± 174	−2

*Note*: Data are from all subjects (*n* = 6). Pre and post volumes are cm^3^. *p* > 0.05 for all muscles from pre‐ to post‐training.

** *p* ≤ 0.1 pre‐ to post‐training.

**FIGURE 3:**
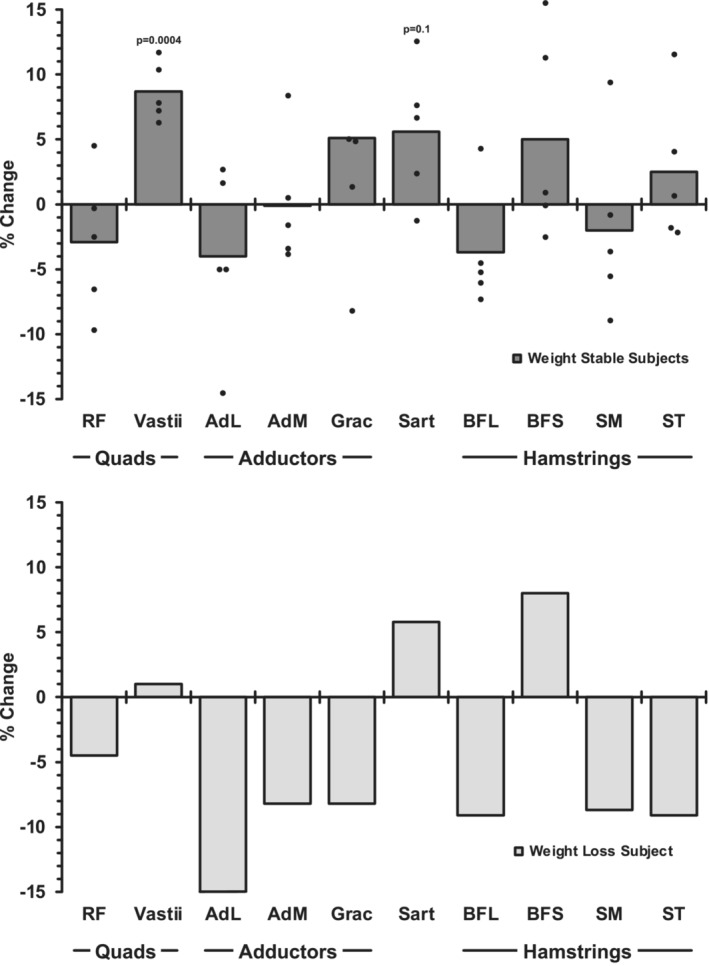
Change in muscle volume of the 10 upper leg muscles in response to the 12 weeks of aerobic cycle exercise training in the five individuals that were weight stable during the training (−1% to +3% from pretraining body weight) (top) compared with the one individual that lost weight (−9%) (bottom). Mean and individual data are presented for the weight‐stable subjects. For scaling purposes, one individual (+22%) was not included for the gracilis muscle. AdL, adductor longus; AdM, adductor magnus; BFL, biceps femoris long head; BFS, biceps femoris short head; Grac, gracilis; RF, rectus femoris; Sart, sartorius; SM, semimembranosus; ST, semitendinosus; Vastii, vastus lateralis, vastus intermedius, vastus medialis; Quads, Quadriceps.

**FIGURE 4:**
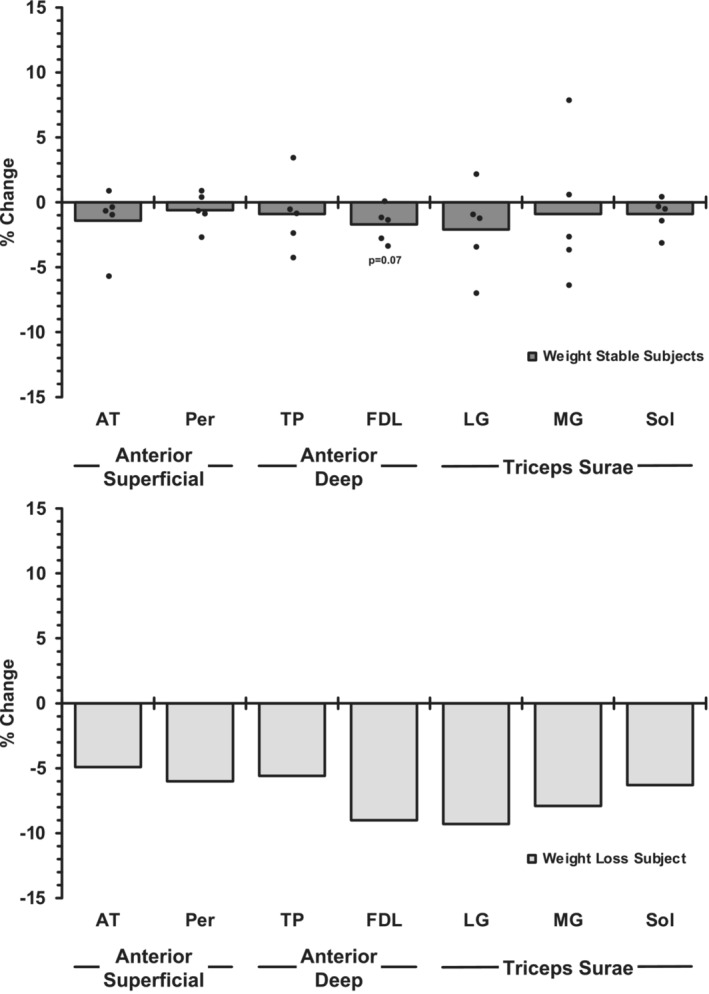
Change in muscle volume of the seven lower leg muscles in response to the 12 weeks of aerobic cycle exercise training in the five individuals that were weight stable during the training (−1% to +3% from pretraining body weight) (top) compared with the one individual that lost weight (−9%) (bottom). Mean and individual data are presented for the weight‐stable subjects. AT, anterior tibial muscles; FDL, flexor digitorium longus; LG, lateral gastrocnemius; MG, medial gastrocnemius; Per, peroneals; Sol, soleus; TP, tibialis posterior. *p* > 0.05 pre‐ to post‐training for all muscles.

**FIGURE 5:**
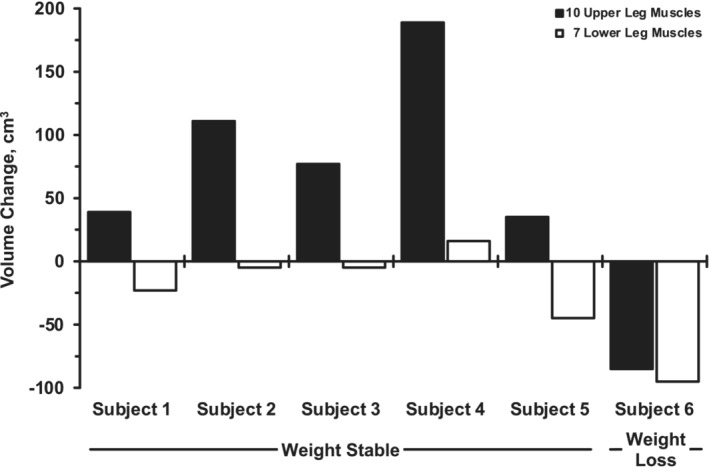
Individual subject changes in muscle volume of the 10 upper leg and seven lower leg muscles in response to the 12 weeks of endurance training in the five individuals that were weight stable during the training (−1% to +3% from pretraining body weight) compared with the one individual that lost weight (−9%). The volume change of each muscle within the upper and lower leg segments were summed together to get the total for each subject. *p* = 0.03 for the *n* = 5 group pre‐ to post‐training for the upper leg muscles (+4 ± 3%). *p* = 0.29 for the *n* = 5 group pre‐ to post‐training for the lower leg muscles (−1 ± 2%).

The weight‐stable subjects compared to the individual that lost a substantial amount of body weight provided some interesting insights (Figures 3, 4, 5). The hypertrophy of the vastii in the weight‐stable subjects (+9%, *p* ≤ 0.05) was mostly eliminated in the weight‐loss subject (+1%). However, the weight loss did not seem to prevent the sartorius (weight stable: +6%, weight loss: +6%) or the biceps femoris short head from hypertrophying (weight stable: +5%, weight loss: +8%). All 7 other muscles of the upper leg atrophied a substantial amount in the weight‐loss subject (−5% to −15%), which corresponds to the muscles that did not change with exercise training in the weight‐stable subjects. For the lower leg muscles, the trend of the training response in all 7 muscles in the weight‐stable subjects (−1% to −2%) was exaggerated in the weight‐loss subject (−5% to −9%). When the volume changes (cm^3^) were summed up the weight‐stable subjects increased (*p* ≤ 0.05) volume of the 10 upper leg muscles, while the total volume of the 7 lower leg muscles was unchanged (*p* > 0.05) (Figure 5). This was not the case for the weight‐loss subject, as he had a substantial loss of total muscle volume of the upper and lower legs.

## 4 DISCUSSION

We have had a long‐standing interest in understanding the influence of specific exercise modes counteracting skeletal muscle atrophy associated with aging and long‐duration microgravity exposure, which generally resembles accelerated skeletal muscle aging (Carrithers et al., 2007; Harber et al., 2012; Harber, Crane, et al., 2009; Harber, Konopka, et al., 2009; Lester et al., 2013; Raue et al., 2012; Slivka et al., 2008; Trappe, 2009; Trappe et al., 2000, 2002, 2009, 2011; Trappe, Burd, et al., 2007; Trappe, Creer, et al., 2007; Trappe, Godard, et al., 2001; Trappe, Raue, & Tesch, 2004; Trappe, Trappe, et al., 2001, 2004). Interestingly, the aging literature is replete of information that is muscle‐specific (Naruse, Trappe, & Trappe, 2023). The quadriceps are commonly studied in aging investigations because of the relative ease in completing imaging, isolated functional measurements, and biopsy‐derived measurements on this muscle group (Naruse, Trappe, & Trappe, 2023). As expected in the current investigation, the vastii muscles were highly responsive to the cycle exercise training (Gregor et al., 1991; Harber et al., 2012; Hug & Dorel, 2009; Konopka & Harber, 2014). The sartorius and the biceps femoris short head of the hamstrings muscles were also responsive, and suggests these muscles are highly engaged in the cycle exercise training performed by the older men. In addition, there are some data to suggest that the lower leg muscles are not highly involved in the muscle activity required to pedal a stationary cycle (Gondoh et al., 2009; Lester et al., 2013). The muscle volume data of the seven muscles of the lower limb studied in the current investigation support these findings. This is important because these muscles of the lower leg are involved in common daily activities like walking and overall stability and balance (Bavdek et al., 2018; Ericson et al., 1986; Oi et al., 2003; Winter & Yack, 1987), which is impaired in older adults (Boyer et al., 2023; Larsson et al., 2019). We recognize that the lack of muscle hypertrophy in response to training, especially aerobic exercise, does not necessarily mean a given muscle is uninvolved with that activity or has not adapted at some level. Indeed, the aforementioned metabolic and EMG studies suggest that some of the muscles that did not hypertrophy in the upper and lower legs of the older individuals in the current study may have undergone beneficial adaptations. However, in the context of age‐related muscle atrophy and the associated loss of muscle function, understanding the impact of common exercise modes on hypertrophy in a muscle‐specific fashion is important to optimize exercise programs for older adults. It appears that other specific exercises are needed for the rectus femoris and adductors, certain hamstrings, and the anterior and posterior lower leg muscles to augment the beneficial effects of cycling exercise for older adults.

The current findings add to other imaging studies, although limited in number, that have measured muscle volume or cross‐sectional area of some lower limb muscles in response to aerobic exercise training (Ema et al., 2016; Konopka & Harber, 2014). While studies in older individuals undergoing aerobic cycle exercise training have shown quadriceps (no separation of the rectus femoris and vastii muscles) hypertrophy similar to that reported in the current study, this finding is not universal (Konopka & Harber, 2014). Relatively lower training intensity, frequency, and duration seem to explain the lack of muscle mass gains with aerobic training, independent of age (Konopka & Harber, 2014). Unfortunately, the available data on older individuals does not provide insight into any other muscle‐specific changes of the upper or lower leg in response to aerobic exercise training in any mode. There are data from middle‐aged women (average 51 years) on some upper leg muscle‐specific responses to 12 weeks of aerobic cycle training (3 sessions per week; 10 min warm up, 40 min at 55%–85% maximal heart rate, 10 min cooldown each session) (Hudelmaier et al., 2010). This investigation showed the quadriceps (+4%) and sartorius (+5%) hypertrophied, while the hamstrings (0%) and adductors (+1%) were unchanged in response to the 3‐month aerobic training program. While specific quadriceps, hamstrings, and adductor muscles were not delineated, these data align with the findings from the current investigation. Interestingly, they also studied a similar group of women completing a 12‐week resistance exercise program and showed similar hypertrophy of the quadriceps (+3%) and sartorius (+4%) as observed with the aerobic training program (Hudelmaier et al., 2010). The ability of properly structured aerobic exercise to stimulate similar muscle mass gains as resistance exercise has been highlighted previously (Konopka & Harber, 2014). Finally, imaging data of the upper and lower leg of astronauts on extended duration spaceflights on the International Space Station show that moderate‐intensity aerobic exercise is relatively ineffective at completely preventing microgravity‐induced atrophy of the posterior lower leg muscles (i.e., soleus and gastrocnemius) (Alkner & Tesch, 2004; Fitts et al., 2010, 2013; Gopalakrishnan et al., 2010; Trappe et al., 2009; Trappe, Burd, et al., 2007). These findings are in agreement with the lack of change in the seven lower leg muscles in the current investigation.

Longitudinal and cross‐sectional studies in younger individuals also provide comparative data to consider. Akima et al. (1997), reported hypertrophy of the biceps femoris short head (+5%) after 6 weeks of sprint cycle training. They also reported an average decrease in the biceps femoris long head (−4%) and an average increase in the sartorius (+5%), although these responses were nonsignificant. Factoring in the relatively short duration of the training, these findings in the biceps femoris short and long heads, and the sartorius are consistent with the current study findings (Figure 3; Table 3). They also showed no change in the semitendinosus (+2%), semimembranosus (−3%), and gracilis (+1%), which are all consistent with the current findings. Hug et al. (2006), compared professional road cyclists to controls and showed 25% larger sartorius muscles in the road cyclists, with no difference in the rectus femoris, semitendinosus, semimembranosus, and gracilis. All of these findings align with the current results. Ema et al. (2016), also have compared experienced cyclists with controls and found some similar muscle differences (or lack thereof) with cycle training as we report here. The vastii and biceps femoris short head were larger in the cyclists and the rectus femoris, semimembranosus, and gracilis were not different between groups. These same types of responses were observed in the experienced cyclists when they were studied before and after a 6‐month cycle training season. While not all of the data from these studies in younger subjects support the current findings (Akima et al., 1997; Ema et al., 2016; Hug et al., 2006), there does seem to be general support for the responses (or lack thereof) we reported in the upper leg muscles of the older men in the current study. However, it should also be considered that competitive cyclists are likely to engage in other forms of training (e.g., resistance training, running). Lastly, there does not appear to be any imaging data from young individuals to provide insight into the responsiveness of the lower limb musculature to aerobic cycle exercise training.

The data from the subject that lost weight during the exercise training supports the idea that the vastii, sartorius, and biceps femoris short head are the most responsive to the cycle exercise stimulus (Figure 3), as these were the only upper leg muscles that did not atrophy in response to the aerobic exercise training in the presence of the catabolic weight loss environment. It is interesting to note that the vastii, the muscles that are highly involved with cycling, did not hypertrophy (+1%) compared to the weight‐stable group (+9%), while the sartorius and biceps femoris short head hypertrophied as much or more than the weight‐stable group (Figure 3). The data from the lower leg, where all seven muscles atrophied to a greater degree than the weight‐stable group (Figure 4), further reinforce the idea that the lower leg muscles were likely not engaged to a great degree in the cycle exercise training. Overall, 15 of the 17 muscles studied in the subject that lost weight responded “negatively” to the aerobic training stimulus compared to the weight‐stable group, confirming the magnitude of the catabolic signals, which resulted in some muscles atrophying fivefold more than the weight‐stable group (Figures 3 and 4). This supports the well‐understood idea that weight loss can compromise lean mass, especially in older individuals (Miller & Wolfe, 2008; Villareal et al., 2017). While the exercise may have played a role in preserving some muscle mass, there was a net loss of both upper and lower leg muscle mass in the weight loss subject (Figure 5) that likely compromised the overall beneficial effects of the exercise training. If weight loss is planned, exercise may help offset the undesired loss of muscle mass (Miller & Wolfe, 2008; Villareal et al., 2017), but the current data suggest strategic, muscle‐specific exercises need to be considered to prevent an exacerbation of the age‐related loss of muscle mass.

### 4.1 Future directions and limitations

The current findings and existing literature should be considered in the context of providing more exercise information for older individuals to have more functional and independent lives. Guidelines from key organizations recognizing exercise as a major tool in combating declines in skeletal muscle and overall health with aging suggest a combination of aerobic and resistance exercises (ACSM, 2021; Piercy et al., 2018). The current findings support this, with specific focus needed for the rectus femoris and adductors, three of the four hamstrings, and all of the lower leg muscles in individuals that want to include cycling exercise in their training plan. Shorter duration, higher‐intensity cycling exercise has been shown to be beneficial for skeletal muscle and cardiovascular health (Gibala & MacInnis, 2022), which corresponds to the previously mentioned data that the calf muscles are more metabolically active in higher‐intensity cycle exercise (Lester et al., 2013). Walking and running have also been shown to substantially engage the posterior lower leg muscles (Coggan et al., 1992; Costill et al., 1974; Ericson et al., 1986; Fujimoto et al., 2000; Harber, Crane, et al., 2009; Oi et al., 2003; Winter & Yack, 1987). Thus, resistance and or aerobic exercise that targets the muscles other than the vastii and sartorius needs to be investigated and incorporated in training regimens for older individuals that primarily use cycling exercise for health benefits.

There are few methodological approaches that can be used to address our overall research interest in this area. Future studies could use other MRI or CT approaches to better understand muscle composition (Goodpaster et al., 2023) and how that may vary among muscles (Naruse, Fountain, et al., 2023), both in response to aging and exercise training. Even more advanced imaging and metabolic studies could be considered, although the radioactivity may be a limitation (Fujimoto et al., 2000; Gondoh et al., 2009; Oi et al., 2003). While we have experience with biopsies of the vastus lateralis, soleus, and gastrocnemius (Dickinson et al., 2010; Harber et al., 2012; Harber, Crane, et al., 2009; Luden et al., 2010; Trappe et al., 1995, 2009, 2011; Trappe, Raue, & Tesch, 2004; Yang et al., 2005), accessing the larger number of lower limb muscles for biopsy sampling is not a realistic consideration. Future studies could also consider a larger sample size. Our post‐hoc sample size estimates (Table S1; https://doi.org/10.6084/m9.figshare.23509494) suggest a modest increase in the number of subjects to ~30 to 40 would have resulted in detecting a training response in only one additional muscle in the upper leg (rectus femoris atrophy) beyond what was observed at the *p* ≤ 0.05 or *p* = 0.1 level. However, it is possible significant atrophy of four more lower limb muscles would have been observed (Table S1; https://doi.org/10.6084/m9.figshare.23509494). Of course, the cost and resources to study this many additional subjects would be substantially higher given the time commitment for the subjects and staff implementing the 12 weeks of exercise training, as well as the MRI acquisition and analysis, which requires a relatively large time commitment per subject. A limitation of the current study is the focus on only men and understanding the muscle‐specific response in women also needs to be considered. It has been shown that women have a relatively more robust hypertrophic response of the quadriceps following cycle exercise training (Harber, Konopka, et al., 2009). As a result, the other muscles of the upper and lower leg in women may respond differently as well.

In summary, this imaging investigation of 17 muscles of the upper and lower leg in response to 3 months of aerobic exercise training in older men adds to our understanding of aging and skeletal muscle health. This information can be incorporated into the planning of exercise programs for older adults. Future studies can also be planned in the context of the current findings to better understand muscle‐specific adaptations to aging and exercise.

## AUTHOR CONTRIBUTIONS

All authors conceived and designed the research, analyzed the data, interpreted results of experiments, edited and revised the manuscript, and approved final version of the manuscript. MN, ARK, MPH, and TAT performed experiments. MN, CSV, and TAT prepared figures. MN and TAT drafted the manuscript.

## FUNDING INFORMATION

This study was supported by NIH Grant AG032127 and NASA Grant NNJ06HF59G.

## CONFLICT OF INTEREST STATEMENT

No conflicts of interest, financial or otherwise, are declared by the authors.

## Supporting information


Table S1.

## Data Availability

Data will be made available by the authors upon reasonable request.
