# Supplementary material for: Cycle exercise training and muscle mass: A preliminary investigation of 17 lower limb muscles in older men
Source: Physiol Rep. 2023 Aug 22;11(16):e15781. doi: 10.14814/phy2.15781 (PMC10442866; doi:10.14814/phy2.15781)
Supplement: Supplementary file 1 — Table S1. [file PHY2-11-e15781-s001.pdf]

Table S1. Number of subjects required to show a significant response to training with the difference (δ) and standard deviation (σ) observed in the current investigation, with an $\alpha=0.05$ and a $\beta=0.2$ (i.e., power of 0.8, 80%).

Muscle	Number of Subjects
**Upper Leg**	
Rectus Femoris	33
Vastii	3
Adductor Longus	23
Adductor Magnus	2249
Gracilis	85
Sartorius	12
Biceps Femoris – Long Head	19
Biceps Femoris – Short Head	24
Semimembranosus	170
Semitendinosus	60
**Lower Leg**	
Anterior Tibial Muscles	26
Peroneals	43
Tibialis Posterior	183
Flexor Digitorum Longus	10
Lateral Gastrocnemius	25
Medial Gastrocnemius	152
Soleus	17

Data from the n=5 weight stable subjects were used for the calculations.
